# Anti-Stress Effects of Carnosine on Restraint-Evoked Immunocompromise in Mice through Spleen Lymphocyte Number Maintenance

**DOI:** 10.1371/journal.pone.0033190

**Published:** 2012-04-12

**Authors:** Yi-Fang Li, Rong-Rong He, Bun Tsoi, Xiao-Di Li, Wei-Xi Li, Keiichi Abe, Hiroshi Kurihara

**Affiliations:** 1 Institute of Traditional Chinese Medicine and Natural Products, Jinan University, Guangzhou, China; 2 BRAND'S Foundation Health Science Research Centre, Jinan University, Guangzhou, China; Université de Montréal, Canada

## Abstract

Carnosine (β-alanyl-L-histidine), a naturally occurring dipeptide, has been characterized as a putative neurotransmitter and serves as a reservoir for brain histamine, which could act on histaminergic neurons system to relieve stress-induced damages. However, understanding of the role of carnosine in stress-evoked immunocompromise is limited. In this study, results showed that when mice were subjected to restraint stress, spleen index and the number of spleen lymphocytes including Natural Killer (NK) cells were obviously decreased. Results also demonstrated that restraint stress decreased the cytotoxic activity of NK cells per spleen (LU_10_/spleen) while the activity of a single NK cell (LU_10_/10^6^ cells) was not changed. However, oral administration of carnosine (150 and 300 mg/kg) increased spleen index and number of spleen lymphocytes (including NK cells), and elevated the cytotoxic activity of NK cells per spleen in restraint-stressed mice. These results indicated that carnosine ameliorated stress-evoked immunocompromise through spleen lymphocyte number maintenance. Carnosine was further found to reduce stress-induced elevation of plasma corticosterone level. On the other hand, results showed that carnosine and RU486 (a glucocorticoids receptor antagonist) treatment prevented the reduction in mitochondrion membrane potential and the release of mitochondrial cytochrome c into cytoplasm, increased Bcl-2/Bax mRNA ratio, as well as decreased terminal deoxynucleotidyl transferase-mediated dUTP-biotin nick end labeling (TUNEL)-positive cells in spleen lymphocytes of stressed mice. The results above suggested that the maintenance of spleen lymphocyte number by carnosine was related with the inhibition of lymphocytes apoptosis caused by glucocorticoids overflow. The stimulation of lymphocyte proliferation by carnosine also contributed to the maintenance of spleen lymphocyte number in stressed mice. In view of the elevated histamine level, the anti-stress effects of carnosine on restraint-evoked immunocompromise might be via carnosine-histamine metabolic pathway. Taken together, carnosine maintained spleen lymphocyte number by inhibiting lymphocyte apoptosis and stimulating lymphocyte proliferation, thus prevented immunocompromise in restraint-stressed mice.

## Introduction

Carnosine (β-alanyl-L-histidine), a naturally occurring dipeptide, is mainly distributed in the brain, muscles, and other innervated tissues [1], [2]. Numerous studies have shown that long-lived cells, such as neurons and myocytes, contain high level of carnosine [3]. Many biological effects of carnosine have been reported. It has been shown to prevent bleomycin-induced lung injury [4], inhibit the growth of neoplastic cells [5], delay diabetic deterioration [6], [7], contribute to the improvement of Alzheimer's disease (AD) [8], and possess anti-aging activities [3], [9], [10]. Earlier reports also indicated that carnosine supplementation could regulate immune function, such as extending the lifespan of human CD4^+^ T cells [11], modulating immune response of rabbits against Schistosoma mansoni antigens and regulating the functions of neutrophils [12], [13]. Underlying mechanism of these effects was suggested to be related with the antioxidative property of carnosine [14]. However, recent studies found that carnosine could exert anti-stress effects through histamine metabolic pathway [15]. Carnosine can be degraded by carnosinase and the resultant histidine is then converted into histamine by histidine decarboxylase [16]. Earlier studies indicated that histamine plays an important role in the alleviation of stress-caused adverse effects [17].

Stress is a familiar aspect of life, being a stimulant for some individuals, but a burden for many others. Stress has long been suspected to play a role in the etiology of many diseases, and numerous studies have shown that stress affects the secretion of neuroendocrine hormones [18], particularly glucocorticoids, to suppress immune system [19], and cause acute organ dysfunction [20]. The reduction of spleen lymphocyte number is an important implication for stress-induced immunocompromise [21]. It may result in disordered homeostasis of immune function which will lead to cancer, neurodegenerative or autoimmune diseases [22]. Previously, carnosine was indicated to possess immune regulation and anti-stress effects [11], [12], [13], [15]. However, effect of carnosine on stress-induced immunocompromise remains unclear. Therefore, in the present study we employed restraint, a common stress-causing factor, to investigate the protective effects of carnosine against stress-induced immunocompromise in mice.

## Results

### Carnosine increased spleen index and spleen lymphocyte number in restraint-stressed mice

As shown in **Table 1**, restraint stress significantly reduced spleen index and spleen lymphocyte number by about 52% and 61% respectively (p<0.01). However, oral administration of carnosine (150, 300 mg/kg) significantly recovered spleen index and lymphocyte number in stressed mice (p<0.05 or p<0.01).

**Table 1:** Effect of carnosine on spleen index and spleen lymphocyte number in restraint-stressed mice.

Group	Spleen index (%)	Spleen lymphocyte number (10^7^)
Normal	0.23±0.16	16.9±3.6
Model (Restraint)	0.11±0.07##	6.6±2.1##
Restraint+Carnosine 150	0.19±0.05*	13.3±2.9**
Restraint+Carnosine 300	0.21±0.11**	14.3±1.8**

On the 7^th^ day of the experiment, all mice except normal control were physically restrained in a 50 ml polypropylene centrifuge tube with holes for 18 h. Spleens were collected from mice and weighted. Spleen index was expressed as spleen weight (g) over body weight (g). Spleens were disrupted with a grinder. After debris separation and erythrocytes lysis, spleen lymphocytes were obtained and counted. The results represent mean ± S.E.M. obtained from 10 animals in each group. The significance of differences from normal group is at

## p<0.01, and from model group at

* p<0.05,

** p<0.01, as determined by ANOVA analysis.

### Carnosine elevated the cytotoxic activity of NK cells per spleen by increasing NK cell number in restraint-stressed mice

As NK cells are important population of lymphocytes and are pivotal players in immune responses against pathogens and tumors. We evaluated the effects of carnosine on NK cell number and cytotoxic activity in spleen by flow cytometry. As shown in Table 2, restraint stress obviously reduced NK cell number (p<0.01). Moreover, results also showed that restraint stress had no obvious effect on the cytotoxic activity of a single NK cell (LU10/10^6^ cells), while NK cell activity per spleen (LU10/spleen) was remarkably suppressed by restraint stress (p<0.01). However, oral administration of carnosine (150, 300 mg/kg) significantly elevated spleen NK cell number (p<0.01), and improved the suppressed NK cell activity per spleen in stressed mice (p<0.05 or p<0.01).

**Table 2:** Effects of carnosine on NK cell number and cytotoxic activity in spleen lymphocytes of restraint-stressed mice.

Group	NK cell number (10^6^)	LU_10_/10^6^ cells	LU_10_/spleen
Normal	2.12±0.75	0.134±0.019	18.46±3.14
Model (Restraint)	0.92±0.21##	0.152±0.022	9.41±2.21##
Restraint+Carnosine 150	1.85±0.35**	0.137±0.026	13.52±3.12*
Restraint+Carnosine 300	1.96±0.42**	0.141±0.014	17.46±3.36**

Spleen lymphocyte samples containing 1×10^6^ cells were stained with anti-CD3 (FITC)/anti-NK1.1 (PE) antibody to assess the percentage of NK cells (NK^+^CD3^−^) by flow cytometry. NK cell number was calculated according to NK cell percentage and total spleen lymphocyte number. In the determination of NK cell cytotoxic activity, target cells were YAC-1 cells. YAC-1 cells, stained with DiO, were mixed with freshly isolated spleen lymphocytes and incubated. Then PI was added to the mixtures. After incubation, NK cell activity was determined by flow cytometry. The results represent mean ± S.E.M. obtained from 10 animals in each group. The significance of differences from normal group was at

## p<0.01, and model group at

* p<0.05,

** p<0.01, as determined by ANOVA analysis.

### Carnosine reduced plasma corticosterone level in restraint-stressed mice

**Figure 1:**
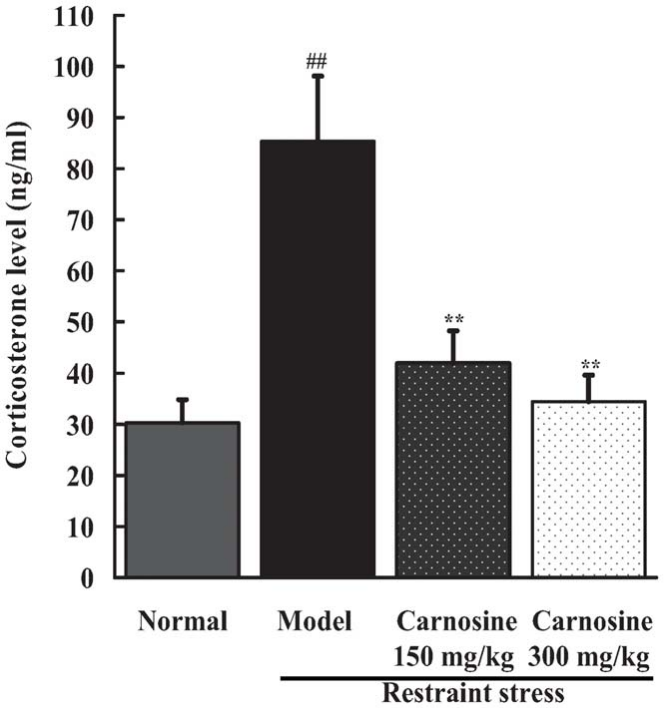
Effect of carnosine on plasma corticosterone level of restraint-stressed mice. Blood containing heparin was centrifuged to obtain plasma. Corticosterone was extracted from plasma by acetic ether, and its content was determined by HPLC with a UV detector at 254 nm. Data represented mean ± S.E.M. obtained from 10 animals in each group. The significance of differences from normal group is at ^##^p<0.01, and from model group at ^**^p<0.01.

Glucocorticoids overflow is an indicator for the activation of hypothalamic-pituitary-adrenal axis (HPA axis) caused by stress. Plasma corticosterone level was evaluated by high-performance liquid chromatography (HPLC) at 254 nm. As shown in Figure 1, restraint stress induced a 2-fold increase in plasma corticosterone level when compared with normal control (p<0.01). However, oral administration of carnosine (150, 300 mg/kg) obviously decreased corticosterone level by 51% and 60% respectively in restraint-stressed mice (p<0.01).

### Carnosine elevated brain histamine levels in restraint-stressed mice

We investigated the effect of carnosine on histamine levels in brain regions by HPLC with an electrochemical detector (ECD). As shown in Table 3, the basal histamine level in normal mice was 110.39±15.17, 89.81±17.95, and 62.07±10.45 pmol/g tissue in hypothalamus, hippocampus and cortex, respectively. When mice were exposed to restraint stress for 18 h, the average histamine level was significantly increased to 345.96±23.12, 149.60±21.24, and 168.49±15.21 pmol/g tissue in hypothalamus, hippocampus and cortex, respectively (p<0.01), indicating histamine play an important role in stress response. On the other hand, when compared with model group, oral administration of carnosine (150 and 300 mg/kg) markedly elevated histamine levels in brain regions of stressed mice (p<0.01).

**Table 3:** Effects of carnosine on brain histamine concentration (pmol/g tissue) in restraint-stressed mice.

Group	Hypothalamus	Hippocampus	Cortex
Normal	110.39±15.17	89.81±17.95	62.07±10.45
Model (Restraint)	345.96±23.12##	149.60±21.24##	168.49±15.21##
Restraint+ Carnosine 150	409.85±30.53**	218.23±35.42**	244.32±18.03**
Restraint+ Carnosine 300	530.08±43.18**	291.77±25.83**	322.75±29.58**

Brain regions including cerebral cortex, hippocampus and hypothalamus were rapidly dissected and homogenized in PBS containing 3% perchloric acid. Histamine level in brain regions was determined by HPLC-ECD. The results represent mean ± S.E.M. obtained from 10 animals in each group. The significance of differences from normal group was at

## p<0.01, and model group at

** p<0.01, as determined by ANOVA analysis.

### Carnosine inhibited the decrease in mitochondria membrane potentials (*ΔΨm*) of spleen lymphocytes in restraint-stressed mice

**Figure 2:**
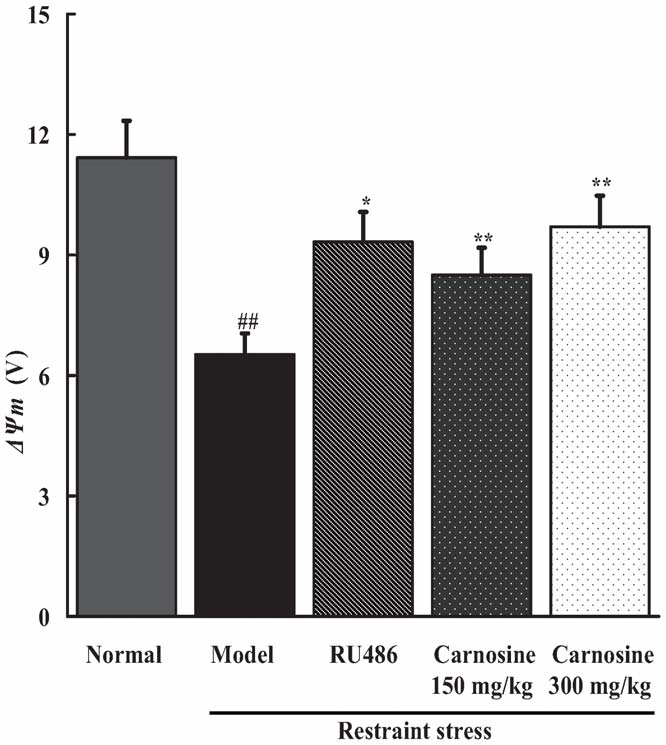
Effect of carnosine on mitochondria membrane potentials of spleen lymphocytes in restraint-stressed mice. Spleen lymphocyte samples containing 5×10^5^ cells were stained with rhodamine-123. After incubation and wash, cells were resuspended in PBS. *ΔΨm* was determined using a flow cytometer. Data represented mean ± S.E.M. obtained from 10 animals in each group. The significance of differences from normal group is at ^##^p<0.01, and from model group at ^*^p<0.05, ^**^p<0.01.

One of the best indicators of mitochondria function is the *ΔΨm*. If mitochondrial membrane is destructed, *ΔΨm* will be reduced. The *ΔΨm* of spleen lymphocytes was measured by flow cytometry. As shown in Figure 2, a huge decrease of fluorescence intensity in restraint stress group was observed when compared with normal control group (p<0.01). Data also demonstrated that RU486, a glucocorticoids receptor antagonist, could elevate the fluorescence intensity in stressed mice (p<0.05). Besides, oral administration of carnosine (150, 300 mg/kg) to stressed mice significantly prevented the decrease of *ΔΨm* (p<0.01).

### Carnosine prevented the leakage of mitochondrial cytochrome c into cytoplasm in spleen lymphocytes of restraint-stressed mice

The decrease in *ΔΨm* implies an alteration of mitochondrial membrane structure, which might result in the release of cytochrome c into cytoplasm. The accumulation of cytochrome c in cytoplasm will activate caspase and eventually lead to cell apoptosis. As shown in Table 4, when compared with normal control, the content of cytochrome c was obviously decreased in mitochondria and increased in cytoplasm in restraint stress group (p<0.01). RU486 could inhibit the leakage of mitochondrial cytochrome c into cytoplasm (p<0.01) in stressed mice. Besides, oral administration of carnosine (150, 300 mg/kg) to stressed mice also showed the effect on preventing leakages of mitochondrial cytochrome c into cytoplasm (p<0.01).

**Table 4:** Effect of carnosine on cytochrome c (Cyt c) contents in mitochondria and cytoplasm in restraint-stressed mice.

Group	Cytoplasm Cyt c (nmol/mg)	Mitochondria Cyt c (nmol/mg)
Normal	18.73±1.06	157.52±14.31
Model (Restraint)	46.42±5.23##	109.92±11.28##
Restraint+RU486	22.57±6.21**	143.26±12.14**
Restraint+Carnosine 150	25.16±3.92**	147.69±14.32**
Restraint+Carnosine 300	20.09±2.98*	152.26±9.59**

Spleen lymphocytes samples containing (2×10^6^) were gently lysed with lysis buffer. Lysates were centrifuged to obtain supernatants (cytosolic extracts free of mitochondria) and the pellets (fraction that contains mitochondria). These two fractions were adjusted to 2 mg protein/ml in the buffer. Contents of cytochrome c were determined using difference spectra at the wavelength pairs of 550∼535, 554∼540 and 563∼577 respectively. The results represent mean ± S.E.M. obtained from 10 animals in each group. The significance of differences from normal group is at

## p<0.01, and from model group at

* p<0.05,

** p<0.01, as determined by ANOVA analysis.

### Carnosine decreased GR mRNA level, and increased Bcl-2/Bax mRNA ratio in spleen lymphocytes of restraint-stressed mice

**Figure 3:**
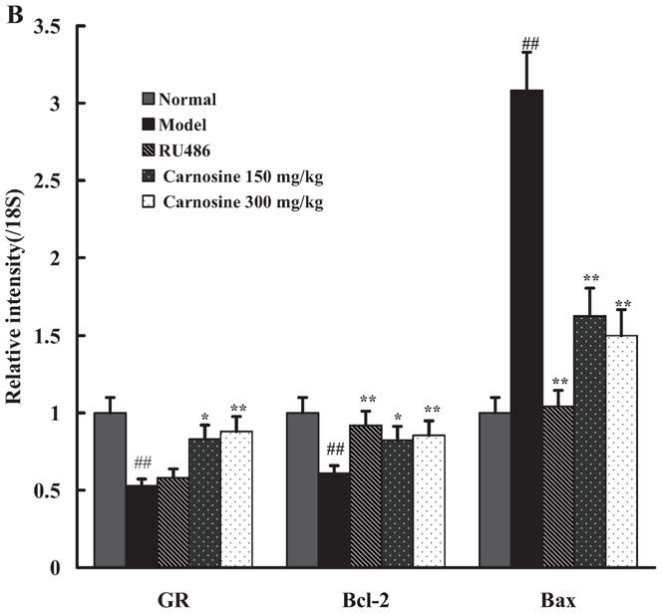
Effect of carnosine on gene expressions of GR, Bax and Bcl-2 mRNAs in spleen lymphocytes of restraint-stressed mice. RT-PCR was employed to determine the effect of carnosine on the expression of GR, Bax and Bcl-2 mRNAs. Relative expression values for each target gene were expressed as a ratio of target gene expression level to 18S expression level. Data represented mean ± S.E.M. obtained from 10 animals in each group. The significance of differences from normal is at ^##^p<0.01, and from model at ^*^p<0.05, ^**^p<0.01.

As shown in Figure 3, restraint stress significantly decreased GR mRNA expression (p<0.01). Moreover, restraint stress markedly up-regulated Bax mRNA expression while down-regulated Bcl-2 mRNA expression (p<0.01), leading to the decrease in mRNA ratio of Bcl-2 to Bax (1.00 vs 0.21). Oral administration of carnosine (150, 300 mg/kg) could evidently reverse the changes in the above gene expressions caused by restraint stress. Besides, RU486 demonstrated an inhibitory effect on stress-induced changes in Bax and Bcl-2 gene expression.

### Carnosine decreased the number of TUNEL-positive cells in spleen of restraint-stressed mice

**Figure 4:**
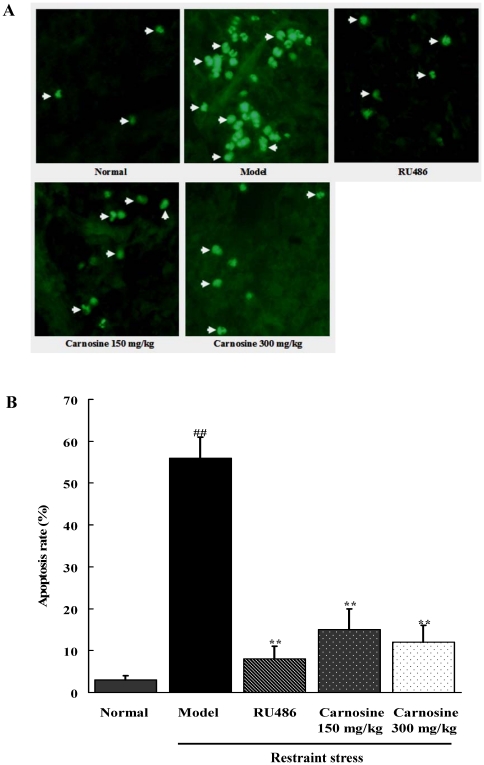
Effect of carnosine on lymphocyte apoptosis confirmed by TUNEL assay. (A) The TUNEL assay was carried out by One Step TUNEL Apoptosis Assay Kit. The images of TUNEL positive cells were captured by a fluorescence microscope (200×). (B) Quantitative result of TUNEL assay was analyzed. Data represented mean ± S.E.M. obtained from 10 animals in each group. The significance of differences from normal is at ^##^p<0.01, and from model at ^**^p<0.01.

There were few TUNEL-positive cells in normal mice (Figure 4). By contrast, the ratio of TUNEL-positive cells increased obviously in restraint-stress mice (p<0.01). However, RU486 and carnosine (150, 300 mg/kg) treatment could evidently decrease the number of TUNEL-positive cells in stressed mice (p<0.01).

### Carnosine had no tendency to inhibit the reduction of spleen lymphocytes (including NK cells) caused by corticosterone treatment *in vitro*

**Figure 5:**
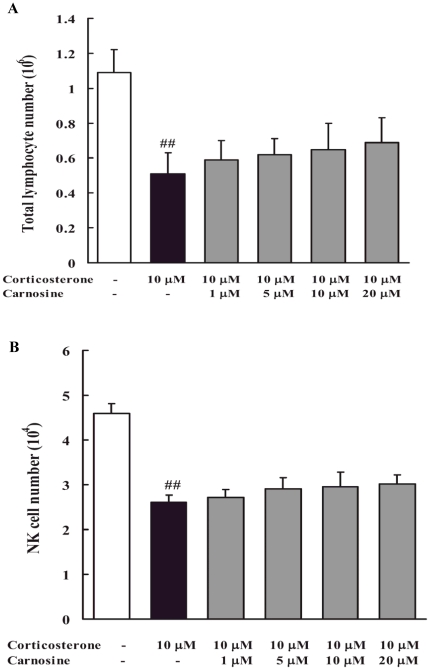
*In vitro* effect of carnosine on the number of spleen lymphocytes (including NK cells) treated with corticosterone. Carnosine had no tendency to inhibit the reduction of total lymphocytes (A) and NK cells (B) induced by corticosterone *in vitro*. Data represented mean ± S.E.M. of triplicate assays. The significance of differences from untreated cells is at ^##^p<0.01.

As shown in Figure 5, total spleen lymphocytes number and NK cell number were significantly decreased by corticosterone (10 µM) treatment *in vitro* (p<0.01), while carnosine (1, 5, 10, 20 µM) had no obvious effect on the number of lymphocytes (including NK cells) treated with corticosterone. These results indicated that carnosine had no direct effect on lymphocyte death induced by corticosterone.

### Carnosine increased spleen lymphocyte proliferation in restraint-stressed mice by administration *in vivo* and treatment *in vitro*

**Figure 6:**
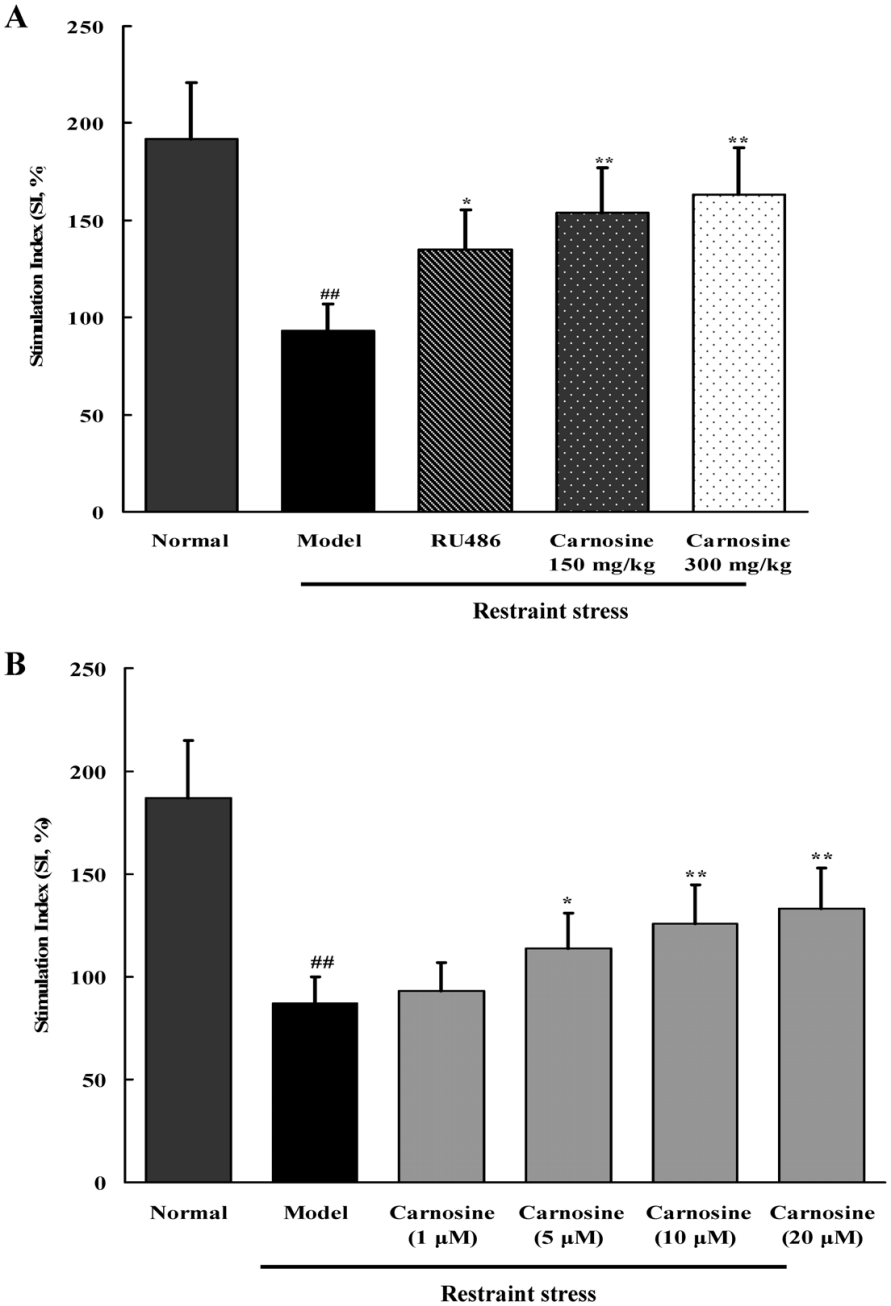
*In vivo* and *in vitro* effects of carnosine on the proliferative response (stimulation index) of spleen lymphocytes stimulated by ConA. (A) Carnosine increased ConA-stimulated lymphocyte proliferation *in vivo*. Data represented mean ± S.E.M. obtained from 10 animals in each group. The significance of differences from normal is at ^##^p<0.01, and from model at ^*^p<0.05, ^**^p<0.01. (B) Carnosine increased ConA-stimulated lymphocyte proliferation by treatment *in vitro*. Data represented mean ± S.E.M. of triplicate assays. The significance of differences from normal is at ^##^p<0.01, and from model at ^*^p<0.05, ^**^p<0.01.

The *in vivo* and *in vitro* effects of carnosine on spleen lymphocyte proliferation were evaluated by MTT method. As shown in Figure 6, restraint stress obviously inhibited the proliferative response (stimulation index) of spleen lymphocytes stimulated with concanavalin A (ConA) (p<0.01). However, carnosine markedly increased ConA-stimulated lymphocyte proliferation in stressed mice whether by administration (150 and 300 mg/kg) *in vivo* or treatment *in vitro* (5, 10, 20 µM).

## Discussion

Previously, studies suggested that the maintenance of spleen lymphocyte number was important for normal immune function [23], [24], [25], [26]. Our research found that restraint stress markedly reduced spleen lymphocyte number and caused spleen atrophy. These results are identical with our previous reports [27], [28]. Results also demonstrated that oral administration of carnosine (150 and 300 mg/kg) to stressed mice could prevent the reduction of spleen lymphocyte number and spleen atrophy.

It is well-known that NK cells are important population of lymphocytes and have key roles in innate immunity and adaptive immunity. They are pivotal players in immune responses against pathogens and tumors [29]. Previous studies have reported that stress suppressed the functions of NK cells, leading to an increased vulnerability of animals to infections or occurrence of malignant tumors [27]. Therefore, we further determined the number and the cytotoxic activity of NK cells in spleen by flow cytometry. Results showed that restraint stress significantly decreased the total number of NK cells in spleen. Besides, we found that restraint stress had no tendency to suppress the cytotoxic activity of a single NK (LU10/10^6^ cells). However, the activity of NK cells per spleen (LU10/spleen) was markedly reduced. These results indicated that the reduction of total NK cell number resulted in the decrease of the activity of NK cells per spleen, which emphasizes the importance of maintaining lymphocyte number to ensure normal immune function. Oral administration of carnosine (150 and 300 mg/kg) obviously increased NK cell number and then elevated the activity of NK cells per spleen in stressed mice, suggesting that carnosine had protective effects against restraint stress-induced immunocompromise in mice by maintaining spleen lymphocytes number.

It is well-known that HPA axis activation and glucocorticoids overflow are key components of the physiological response to stress [30]. Our results showed that restraint stress markedly increased plasma corticosterone level. Previous reports suggested that glucocorticoids overflow triggered spleen lymphocyte apoptosis, which may account for lymphocytes reduction and spleen atrophy [21]. Glucocorticoids-evoked apoptosis is believed to be mediated by glucocorticoids receptor (GR) [22], [31]. GR is activated by overflowed glucocorticoids and translocates into nucleus to regulate some apoptosis-related genes expression [32], among which Bax and Bcl-2 mRNA expression and their corresponding proteins are particularly important [33], 34. Under normal conditions, Bax is present in monomeric form in the cytosol or loosely attached to mitochondrial outer membranes. After activation, Bax combines with mitochondria and becomes a cross-linkable integral membrane protein as a homodimer. Bax may then form channels allowing the release of several apoptosis-related proteins from the mitochondria to propagate the apoptotic pathway [35]. Unlike Bax, Bcl-2 is mainly localized as an integral mitochondrial membrane protein, and forms heterodimers with Bax to prevent mitochondrial changes in apoptosis [36], [37]. Thus the ratio of Bax/Bcl-2 plays an important role in determining cell apoptosis. Moreover, mitochondria have shown to play a key role in glucocorticiods-evoked apoptosis. Alterations in the function of mitochondria could be triggered by the imbalance of Bax/Bcl-2 or other factors and results in a membrane permeability transition (PT) [38]. PT provokes a decline in *ΔΨm* and the release of proapoptotic agents, notably cytochrome c, into the cytoplasm which will activate caspases and eventually cause apoptosis. Our data demonstrated that mRNA ratio of Bcl-2 to Bax was obviously lowered by restraint stress, accompanied with reduced *ΔΨm*, decreased mitochondrial cytochrome c and increased cytoplasm cytochrome c. Further, results of TUNEL experiment showed that restraint stress markedly increased TUNEL-positive cells in spleen. However, RU486, a GR antagonist [31], was found to inhibit these changes in stressed mice. All the above indicated that stress-induced glucocorticoids overflow triggered spleen lymphocyte apoptosis via activating GR and resulted in the reduction of spleen lymphocyte number. Although our results showed that GR mRNA expression was down-regulated in stressed mice, earlier reports indicated that its sensitivity to glucocorticoids and the efficiency of GR-mediated signal transduction were enhanced [32]. In order to confirm the direct effect of glucocorticoids on lymphocytes, spleen lymphocytes were treated with corticosterone (20 µM) *in vitro* and results demonstrated that it obviously decreased the number of lymphocytes (including NK cells). The current findings also showed oral administration of carnosine (150 and 300 mg/kg) to restraint-stressed mice could evidently decrease plasma corticosterone level while up-regulate GR mRNA expression, inhibit the imbalance of Bax/Bcl-2 mRNA expression, elevated *ΔΨm*, prevent the leakage of mitochondrial cytochrome c into cytoplasm, and decrease TUNEL-positive cells in spleen. Theses results demonstrate that the protective effects of carnosine against stress-induced reduction of spleen lymphocyte number correlate with regulating glucocorticoids level to prevent cell apoptosis. However, we did not observe the protective effect of carnosine on the reduction of spleen lymphocytes (including NK cells) caused by corticosterone *in vitro*, indicating that carnosine may not play direct role in protecting lymphocyte from death induced by glucocorticoids.

Previous researches demonstrated that glucocorticoids overflow not only caused lymphocyte apoptosis, but also suppressed lymphocyte proliferation [39], [40]. Results of the present study found that restraint stress obviously inhibited ConA-stimulated spleen lymphocyte proliferation. However, carnosine markedly increased lymphocyte proliferation in stressed mice whether by administration (150 and 300 mg/kg) *in vivo* or treatment *in vitro* (5, 10, 20 µM).

It is reported that carnosine could readily pass through the blood brain barrier (BBB), and acts as the precursor of transmitter of histaminergic neuron system and effectively regulate brain histamine level [16], [41], [42]. The role of brain histamine in regulating stress response has recently been documented [43], [44], and the anti-stress action of exogenous histamine has been demonstrated [17]. Sakai et al. also reported that fluoromethylhistidine (FMH), a histidine decarboxylase inhibitor, reduced the histaminergic nerve metabolism and increased the locomotor activity in rats exposed to stress [45]. However, histamine cannot cross BBB and may be involved in brain inflammation [46]. The infusion of histamine into rat substantia nigra results in an acute inflammatory response manifested by a loss of glial fibrillary acidic protein-immunolabeled astrocytes [47]. Nevertheless, carnosine could serve as a reservoir for histamine with few side effects. Recent studies by Zhu et al. demonstrated that carnosine activated histamine neurons in histidine decarboxylase knock-out mice [48]. Our results showed that carnosine administration significantly elevated brain histamine level in restraint-stressed mice. Taken together, it can be inferred that the anti-stress effect of carnosine on restraint-evoked immunocompromise might be mediated via carnosine-histamine metabolic pathway.

In conclusion, restraint stress triggered responses in HPA axis and caused glucocorticoids overflow, which induced the reduction of spleen lymphocytes number and caused immunocompromise by evoking lymphocyte apoptosis and inhibiting lymphocyte proliferation. Carnosine could maintain spleen lymphocyte number to prevent immunocompromise caused by restraint stress. The mechanism may be mediated via its anti-stress action through carnosine-histamine metabolic pathway. By this mechanism, carnosine inhibited lymphocyte apoptosis and stimulated lymphocyte proliferation to maintain spleen lymphocytes number and prevent immunocompromise caused by stress.

## Materials and Methods

### Ethics Statement

This study was conducted in strict accordance with the “Guide for Care and Use of Laboratory Animals” published by the National Institutes of Health and the ethical guidelines of the International Association for the Study of Pain. The protocol was approved by the Animal Care and Use Committee of the Institute of Traditional Chinese Medicine and Natural Products, Jinan University, China (Permit Number: 20100927).

### Animals and treatment

Seven-week-old male Kunming and C57BL/6 mice were purchased from Guangdong Medical Laboratory Animal Center, Guangzhou, China. All mice were kept in a specific pathogen-free animal room under controlled conditions at the temperature (23±1°C) with a 12-h light-dark cycle (lights on from 06:00 to 18:00). The animals were allowed to acclimatize to the environment for 1 week before experiment. In this study, carnosine was dissolved in drinking water and orally administered to animals at dosages of 150 and 300 mg/kg body weight daily for continuous 7 d [15]. Mice in normal control and restraint stress model groups were administrated with the same volume of water. On the 7^th^ day of the experiment, all mice except normal control were physically restrained in a 50 ml polypropylene centrifuge tube with holes for 18 h [28], [49]. Mice in normal group were allowed to move freely, but neither food nor water was given as restraint-stressed mice. After restraint, mice were weighted and anesthetized by diethyl ether. Blood was obtained by cardiac puncture and, brain and spleen were removed and weighed. Besides, in order to investigate the mechanism responsible for stress-induced reduction of spleen lymphocyte number, an extra RU486 (a glucocorticoids receptor antagonist) group was added in some experiments. RU486 (20 mg/kg) was injected intraperitoneally to mice 30 min before restraint stress. All experiments were conducted with Kunming mice except the determination of NK cell number and the assay of NK cytotoxic activity. L-carnosine and RU486 were purchased from Sigma (St. Louis, USA).

### Cell cultures

YAC-1 tumor cell line, a Moloney virus induced mouse T cell lymphoma of A/SN origin, was obtained from Institute of Health Care Science (Suntory Ltd., Japan). The YAC-1 cell was used to test the NK cytotoxic activity for its noted sensitivity to NK cells. Cell line was cultured in RPMI-1640 containing 10% fetal bovine serum (FBS) at 37°C in a 5% CO_2_ humidified atmosphere before testing.

### Plasma corticosterone assay

Blood containing 100 U/mL heparin was transferred into 1.5 mL centrifuge tubes and plasma was collected after centrifugation at 7 000 rpm for 10 min. Corticosterone was extracted from plasma and quantified by HPLC using a modification of the method reported by Woodward and Emery [50]. Plasma (0.5 mL) was mixed with 30 µL of cortisone solution (0.125 mg/mL methanol-water (60∶40 v/v)) as an internal standard. Steroids were extracted by adding 2 mL of acetic ether and mixing thoroughly. The mixture was immediately centrifuged at 1 500 rpm for 5 min. The organic phase was washed twice with 1 mL of HPLC-grade water and centrifuged. The organic phase was then evaporated at room temperature under nitrogen. The residue was re-dissolved in 100 µL of methanol-water (60∶40 v/v) to measure corticosterone level using HPLC with a UV detector at 254 nm (Hitachi, Japan). The column (5C18, 4.6×100 mm; particle size 5 µm; Waters Corp., Milford, Massachusetts) was equilibrated using HPLC grade acetonitrile-water (38∶72 v/v) at a flow rate of 1 mL/min. Corticosterone and cortisone standards were purchased from Sigma (St. Louis, USA).

### Brain histamine assay

Brain regions including cerebral cortex, hippocampus and hypothalamus were rapidly dissected on ice. The tissues were weighed and soaked in PBS solution containing 3% perchloric acid (1 g tissue/mL). The tissues were homogenized with a homogenizer (IKA Labortechnik, Germany) at maximum setting for 30 s under an ice-cold condition. Then the homogenate was centrifuged at 15 000 rpm for 20 min at 4°C. Supernatant was filtered through a 0.45 µm membrane filter to obtain brain samples. The concentrations of histamine in brain regions were determined by HPLC-ECD, using the derivation method. The system consists of model 582 infusion pump, model 542 automatic sampler, model 5600A CoulArray electrochemical detector (ESA Company, USA). The HPLC was controlled and the data was acquired using CoulArray® software. Brain samples were reacted with the derivate *o*-phthalaldehyde, and analytes were separated on a chromatographic column (Develosil ODS-UG-5, 4.6 mm×150 mm, Nomura Chemical). The mobile phase contained 100 mM Na_2_HPO_4_ (pH 6.8), 13% acetonitrile, and 22% methanol. The flow rate was set to 0.75 mL/min. The temperature of column was maintained at 38°C. Electrochemical detection was designed using two-way electrode potentials (passage I: −150 mV; passage IV: +550 mV). Histamine standard was obtained from Sigma (St. Louis, USA).

### Spleen lymphocytes preparation

The spleens were collected and splenocytes were prepared by disrupting the spleen with a grinder in phosphate-buffered saline (PBS, pH 7.4). After a 10 min centrifugation at 1 500 rpm to separate debris, erythrocytes were lysed using ammonium chloride reagent. The cells were washed twice with PBS and suspended in 0.5 mL of cold RPMI-1640 medium with 10% FBS. Spleen lymphocyte number was determined with a blood-cell counting chamber (Erma, Japan) [28]. The viability of lymphocytes was determined by trypan blue exclusion.

### Determination of NK cell number

Spleen lymphocyte samples containing 1×10^6^ cells in RPMI-1640 medium were treated with selected monoclonal antibodies conjugated with FITC or PE (Beckman, USA). We used the following double-staining combinations: anti-CD3 (FITC)/anti-NK1.1 (PE). Mouse IgG1-FITC and -PE were used as control staining. After 15 min incubation at room temperature in the dark, cells were washed with PBS and resuspended in 0.5 mL of PBS and analyzed using a flow cytometer (Beckman, USA). Usually, 10 000 cells were scanned for each sample, and their results are expressed as the percentage of cells yielding a specific fluorescence in a gated lymphocyte region. NK cell number in each spleen was calculated according to NK cell percentage and total spleen lymphocyte number.

*In vitro* effect of carnosine on NK cell number in spleen lymphocytes treated with corticosterone was also evaluated. In this experiment, spleen lymphocytes were obtained from 5 mice as described above and cultured in RPMI 1640 medium supplemented with 100 U/mL penicillin, 100 µg/mL streptomycin, and 10% heat inactivated FBS. Cells (1×10^6^/well) were cultured in 96-well plates at 37°C in 5% CO_2_. Cells were treated with different concentrations of carnosine (1, 5, 10, 20 µM) and incubated for 24 h, and then added with corticosterone (10 µM) and incubated for 12 h. Cells were collected by centrifugation and resuspended in PBS, followed by staining with antibodies to determine NK cell number. Besides, *in vitro* effect of carnosine on the number of total spleen lymphocytes treated with corticosterone was determined by the 3-(4,5-dimethylthiazol-2-yl)-2,5-diphenyl-tetrazolium bromide (MTT) assay. Briefly, an MTT stock solution (5 mg/mL in PBS) was added to each well (15 µL/well) and incubated for 4 h. The formed formazan was then dissolved by adding 200 µL DMSO per well and shake gently. The absorbance was read on a spectrophotometer at 570 nm. Cell viability was expressed as the percentage of MTT reduction, assuming that the absorbance of control cells was 100%. Total lymphocyte number in each group was calculated according to cell viability.

### Cytotoxic activity of NK cells assay

The experiments employed two fluorescent stains as Piriou [51] reported, 3,3′-dioctadecyloxacarbocyanine perchlorate (DiO, from Sigma) which is stably integrated into the cell membrane and stains the intended cell population homogeneously, and propidium iodide (PI, from Sigma) which passes rapidly through damaged cell membranes and binds with very high affinity to the DNA. NK cell activity was detected with the freshly isolated spleen lymphocytes [28]. Target cells for detection of NK cell cytotoxicity were YAC-1 cells. The YAC-1 cells were maintained in continuous suspension and cultured in the complete culture medium at a concentration of about 1×10^6^ cells/mL at 37°C in a 5% CO_2_ humidified incubator. All cultures were split 24 h before use to ensure that the YAC-1 cells were in an exponential growth phase during the assays. DiO was firstly added to the YAC-1 cells at a concentration of 30 µM, which was incubated for 15 min at 37°C in 5% CO_2_. After labeling, the YAC-1 cells were washed twice, counted and adjusted to 1×10^5^ cells/mL. Mixtures of the stained YAC-1 cells (0.1 mL of 1×10^5^ cells/mL) and spleen lymphocytes (0.1 mL of 1×10^7^, 5×10^6^, and 1.25×10^6^ cells/mL) were incubated at 37°C in a 5% CO_2_ humidified incubator for 4 h. Another mixture of the stained YAC-1 cells (0.1 mL) and the complete culture medium (0.1 mL) were performed in parallel to serve as control for assessment of spontaneously dead YAC-1 cells. Then PI was added to the mixtures at a concentration of 5 mg/mL for 15 min at room temperature. NK cell activity of the above was thus determined. Intact NK cells were non-fluorescent, dead NK cells emitted red fluorescence, living YAC-1 cells exhibited green fluorescence and dead YAC-1 cells were characterized by double (green-red) fluorescence. Flow cytometry was performed with a FACS Epics XL (Beckman, USA) equipped with an argon laser operating at 488 nm. Two parameter dot plots were obtained with Cell Quest software (Beckman, USA). In these plots the abscissa was log scale green fluorescence and the ordinate log scale red fluorescence. In each tested sample, spontaneously dead YAC-1 cells were subtracted from total dead YAC-1 cells and NK cell cytotoxicity was expressed as the percentage of dead YAC-1 cells in total YAC-1 cells. Results were plotted, and the number of cells required to produce 10% specific cytotoxicity (one lytic unit, 1 LU10) was established from the best fit of the curve. The number of LU10 per spleen (LU10/spleen) was thus calculated and used to express final results. Assays for each effector/target cell ratio were performed in triplicate. After incubation for 4 h at 37°C under a 5% CO_2_ atmosphere, lysis of target cells was calculated using formula 1.
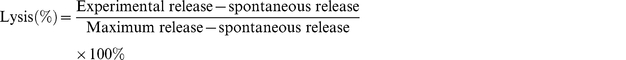
(formula 1)


The maximum release referred to the lysis obtained after adding Triton X-100 (final concentration 1%). Spontaneous release was determined by the incubation of labeled target cells in the absence of effector cells.

### Determination of mitochondria membrane potentials

In this experiment, mitochondria membrane potentials (*ΔΨm*) of spleen lymphocytes were determined using a fluorescent probe, rhodamine-123 (Sigma, USA), a lipophilic cation that accumulates in the mitochondrial matrix in proportion to mitochondrial membrane potential. Spleen lymphocytes suspensions (5×10^5^) were incubated with 10 µM rhodamine-123 at 37°C for 30 min and then thoroughly washed three times with PBS. The fluorescence was determined using a flow cytometer.

### Measurement of cytochrome c contents in mitochondria and cytoplasm

Spleen lymphocytes (2×10^6^) were collected, washed once with PBS and gently lysed for 2 min in 80 µL lysis buffer (250 mM sucrose, 1 mM EDTA, 20 mM Tris-HCl pH 7.2, 1 mM DTT, 10 mM KCl, 1.5 mM MgCl_2_, 5 µg/mL pepstatin A, 10 µg/mL leupeptin, 2 µg/mL aprotinin). Lysates were centrifuged at 12 000 rpm for 10 min to obtain supernatants (cytosolic extracts free of mitochondria) and the pellets (fraction that contains mitochondria). These two fractions were adjusted to 2 mg protein/mL in the buffer. Contents of cytochrome c (Cyt c) were calculated from difference spectra at the wavelength pairs of 550∼535, 554∼540 and 563∼577 respectively (shown in formula 2).
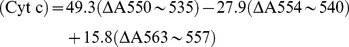
(formula 2)


### Measurement of GR, Bax, and Bcl-2 mRNA expression

Gene expression was determined by quantitative real-time reverse transcription-polymerase chain reaction (RT-PCR) method using Maxima™ SYBR Green/Fluorescein qPCR Master Mix (Fermentas, Hanover, MD, USA) via IQ™5 real-time PCR detection system (Bio-Rad, Hercules, CA, USA). Spleen lymphocytes (5×10^7^) were centrifuged at 6 000 rpm. The upper water layer was removed carefully and cells were added with 1 mL Trizol (Invitrogen, CA) to extract total RNA. Total RNA (3 µg) was reverse-transcribed at 42°C for 1 h in 20 mL reaction mixture containing mouse Moloney leukemia virus reverse transcriptase (Tiangen, Germany) with oligo (dT) primers (Tiangen, Germany) followed by RT-PCR amplification. The mRNA primers for mouse GR (GenBank acc.no. X04435, F: 5′-TGGTGTGCTCCGATGA-3′, R: 5′-AGGGTAGGGGTAAGC-3′, bp: 294), Bax (GenBank acc.no. NM_007527, F: 5′-TCCCACATAACTCCCTCGACA-3′, R: 5′-GGCGAAGCCAGCGAGAAGTCCC-3′, bp: 228), Bcl-2 (GenBank acc.no. NM_009741, F: 5′-CTGGAGGTCTGAAGCG-3′, R: 5′-ATGAATCGGGAGTTGG-3′, bp: 301), and 18S (GenBank acc.no. K01364, F: 5′-AGGGGAGAGCGGGTAAGAGA-3′, R: 5′-GGACAGGACTAGGCGGAACA -3′, bp: 241) were used. The final products were analyzed by Ct method.

### TUNEL assay

The TUNEL method was performed to label 3′-end of fragmented DNA of the apoptotic spleen lymphocytes. Spleens were fleshly removed and frozen in Tissue-Tek OCT Compound (Miles, Elkhart, IN) by immersion in a 2-methylbutane bath on dry ice. 5 µm sections were cut using a Leica CM1900 cryostat, and stained by terminal deoxynucleotidyl transferase-mediated dUTP-biotin nick end labeling (TUNEL) method, using an apoptosis *in situ* detection kit (Wako Pure Chemical, Osaka, Japan), according to the instruction. The FITC-labeled TUNEL-positive cells were imaged under a fluorescent microscopy by using 488 nm excitation and 530 nm emission. After four cortical fields were randomly selected from each section, 100 cells were successively counted for each field by an observer who did not identify the slides. The ratio of TUNEL-positive cell number to the total cell number is shown.

### Spleen lymphocyte proliferation assay

*In vivo* and *in vitro* effects of carnosine on spleen lymphocyte proliferation in stressed mice were evaluated. For the *in vivo* experiment, spleen lymphocytes isolated from mice in normal control group, model group, RU486 treatment and carnosine treatment groups (150 and 300 mg/kg) were cultured in RPMI 1640 medium with 2 mM L-glutamine, 100 U/mL penicillin, 100 µg/mL streptomycin, and 10% heat inactivated FBS. Cells (5×10^5^/well) were cultured in 96-well plates and stimulated with ConA (5 µg/mL) for 48 h. The proliferative response of spleen lymphocytes was determined by MTT assay as previously described [52]. The absorbance of each sample and control (ConA-free medium, with DMSO) was read on a spectrophotometer at 570 nm. Stimulation index (SI) was calculated as formula 3 and expressed as percentage of the control.
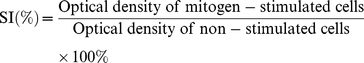
(formula 3)


For the *in vitro* experiment, spleen lymphocytes isolated from normal mice and restraint-stressed mice were cultured in 96-well plates. Cells (5×10^5^/well) were stimulated with concanavalin A (ConA, 5 µg/mL) for 48 h. Then different concentrations of carnosine (1, 5, 10, 20 µM) were added to cells obtained from restraint-stressed mice, while the same volume of medium was added to cells obtained from normal mice. After incubation for 12 h, spleen lymphocyte proliferation was determined by MTT assay and SI was calculated as formula 2.

### Statistical analysis

The data were presented as mean ± S.E.M. Statistical analysis of data was performed using SPSS 13.0 statistical package. One-way analysis of variance (ANOVA) was applied to analyze for difference in data of biochemical parameters among the different groups, followed by Dunnett's significant post-hoc test for pair-wise multiple comparisons. Differences were considered as statistically significant at p<0.05.
